# *Plasmodium vivax* gametocyte infectivity in sub-microscopic infections

**DOI:** 10.1186/s12936-016-1104-1

**Published:** 2016-01-28

**Authors:** Andrés F. Vallejo, Jhon García, Andrés B. Amado-Garavito, Myriam Arévalo-Herrera, Sócrates Herrera

**Affiliations:** Malaria Vaccine and Drug Development Centre (MVDC), Cali, Colombia; Caucaseco Scientific Research Centre (CSRC)/Centro Latino Americano de Investigación en Malaria (CLAIM), Cali, Colombia; Facultad de Salud, Universidad del Valle, Cali, Colombia

**Keywords:** *Plasmodium vivax*, Infectivity, Asymptomatic infections

## Abstract

**Background:**

The use of molecular techniques has put in the spotlight the existence of a large mass of malaria sub-microscopic infections among apparently healthy populations. These sub-microscopic infections are considered an important pool for maintained malaria transmission.

**Methods:**

In order to assess the appearance of *Plasmodium vivax* gametocytes in circulation, gametocyte density and the parasite infectivity to *Anopheles* mosquitoes, a study was designed to compare three groups of volunteers either experimentally infected with *P. vivax* sporozoites (early infections; n = 16) or naturally infected patients (acute malaria, n = 16 and asymptomatic, n = 14). In order to determine gametocyte stage, a quantitative reverse transcriptase PCR (RT-qPCR) assay targeting two sexual stage-specific molecular markers was used. Parasite infectivity was assessed by membrane feeding assays (MFA).

**Results:**

In early infections *P. vivax* gametocytes could be detected starting at day 7 without giving rise to infected mosquitoes during 13 days of follow-up. Asymptomatic carriers, with presumably long-lasting infections, presented the highest proportion of mature gametocytes and were as infective as acute patients.

**Conclusions:**

This study shows the potential role of *P. vivax* asymptomatic carriers in malaria transmission should be considered when new policies are envisioned to redirect malaria control strategies towards targeting asymptomatic infections as a tool for malaria elimination.

**Electronic supplementary material:**

The online version of this article (doi:10.1186/s12936-016-1104-1) contains supplementary material, which is available to authorized users.

## Background

Despite several features being similar among different species of malaria parasites, there are important biological differences between the two most relevant to human infections, *Plasmodium falciparum* and *Plasmodium vivax*. While *P. falciparum* is highly prevalent in the African continent, *P. vivax* is more prevalent in Central Asia, Southeast Asia and the American continent [1]. *P. vivax* infection is characterized by induction of periodical asymptomatic infection or clinical episodes due to spontaneous activation of hypnozoites, dormant parasite liver forms that do not occur in *P. falciparum* infections [2]. In blood circulation, *P. vivax* has a preference for young erythrocytes whereas *P. falciparum* usually invades circulating erythrocytes of any age. Both species develop and multiply asexually inside erythrocytes where a small proportion of the parasite population switches from the asexually replicating to the sexual stage in a process initiated by a differentiation step [3]. While *P. falciparum* is known to differentiate into gametocytes in a relatively late phase of parasite blood development, *P. vivax* appears to produce mature gametocytes significantly earlier, due to the shorter developmental cycle in comparison with *P. falciparum*, which is believed to result in greater transmissibility [4, 5].

Although gametocytes may be present at early stages of an infection, they require a maturation process where factors such as gametocyte density could influence infectivity to mosquitoes [6]. Moreover in areas with low levels of transmission there is a high proportion of infections undetected by microscopy that could be a reservoir for the parasite with unknown gametocyte and infectivity levels [7]. Due to the lack of long-term *P. vivax* in vitro cultures and the lack of suitable animal models, the study of *P. vivax* gametocyte maturation has been limited to the use of parasite samples from naturally infected patients [8–10]. Furthermore, the role of asymptomatic carriers in malaria transmission has not been clearly established [11, 12]. A better understanding of parasite maturation and transmission to mosquitoes would contribute to comprehending the patient-mosquito interplay, which is essential for developing malaria control strategies, such as anti-malarial drugs and vaccines to block malaria transmission [13]. The current study describes the dynamics of *P. vivax* gametocyte production and infectivity in early, acute and asymptomatic infections.

## Methods

### Ethics statement

Samples used in this study were obtained from studies approved by the Institutional Review Board (IRB) of the Malaria Vaccine and Drug Development Centre (MVDC) under the codes CIV-01-042009, CIV 08-102010 and CIV 009. Samples from volunteers were collected anonymously and not linked to the identity of the donor. Written informed consent (IC) was obtained from each volunteer at enrolment. All volunteers were adults aged 18–55 years (Table 1).

**Table 1:** Human subjects

	Early infected volunteers (n = 16)	Symptomatic volunteers (n = 16)	Asymptomatic volunteers (n = 14)
Male (%)	63	75	42.8
Age (%)			
<25	25.0	37.5	14.3
25–34	56.0	37.5	14.3
>34	19.0	25	71.4
Mean	29.7	28.9	36.4
Malaria previous episodes (%)	56.0	93.8	71.4
Mean of malaria episodes	5.1	5.2	3.5
Symptoms (%)			
Chills	69.0	81.5	0
Headache	69.0	81.5	0
Fever	56.0	25.5	0
PCR parasite density			
Mean parasitaemia (parasites/uL)	425.6	5096	13.9
Infectivity to mosquitoes			
Infective samples (%)	0	50	57
Mosquito prevalence (%)	0	57	4.2
Mosquito intensity (mean oocyst)	0	63.5	5.4

### Study sites

Field activities were conducted in two endemic regions of Colombia, Buenaventura and Tierralta. Buenaventura (Department of Valle del Cauca) is the main port of Colombia on the Pacific Coast, located at 7 m asl, with a population of ~400,000 inhabitants and 307 malaria cases reported in 2014 (77.2 % *P. vivax*). Tierralta (Department of Cordoba) is located at 5 m asl in the northern region of the country. In 2014 the town reported 840 malaria cases (94.8 % *P. vivax*).

### Studied population

A total of 46 individuals from early, acute and asymptomatic conditions were studied between January and December 2013 (Table 1). Volunteers were adults aged 18–55 years, male (n = 28) and female (n = 18). The individuals were divided in three groups: early, acute and asymptomatic. Early infected patients (n = 16) were enrolled in a previously reported clinical trial [14]. Asymptomatic (n = 14) *P. vivax* carriers were enrolled by a cross-sectional survey of 180 individuals in Buenaventura. An asymptomatic carrier was defined as an individual without malaria symptoms with either sub-microscopic *P. vivax* infection after one week of follow-up. Patients attending malaria diagnostic facilities (n = 16) in Buenaventura were included in a group denominated ‘acute patients’; all had non-complicated *P. vivax* malaria symptoms and were diagnosed by thick blood smear (TBS) and later confirmed by quantitative PCR (qPCR).

### Patients and sample collection

#### Early infection volunteers

The time line of infectivity and gametocyte maturation in early infections was assessed using samples collected from naïve and semi-immune volunteers participating in a *P. vivax* sporozoite experimental challenge [14]. Sixteen volunteers, 18–55 years of age (seven malaria-naïve and nine previously exposed semi-immune volunteers) were experimentally infected with *P. vivax* sporozoites through the bites of laboratory-reared infected *Anopheles* mosquitoes. Membrane feeding assay (MFA) was performed every 2 days starting at day 7 post-infection until the parasites were detected by microscopy (days 11–13). Kinetics of parasitaemia was measured by qPCR at the same time points as MFA [15]. These volunteers were given curative treatment upon microscopic observation (TBS) of circulating parasites, according to the Colombian Ministry of Health guidelines for malaria treatment, with twice daily dose of primaquine (for *P. vivax,* chloroquine 25 mg/kg provided in three doses and primaquine 0.50 mg/kg daily for 14 days) [16].

#### Asymptomatic carriers

Volunteers (n = 14) participating in a cross-sectional epidemiological survey carried out in June 2012 in La Delfina and Zacarias (two rural areas of Buenaventura, Colombia) with low malaria transmission were selected and enrolled. After a population census, inhabitants of randomly selected houses were asked to supply blood samples to perform TBS and qPCR. At day 7 after PCR parasite detection, a blood sample was collected to perform MFA.

#### Acute malaria patients

Symptomatic volunteers (n = 16) were recruited in malaria diagnosis outpatient clinics of endemic settings in Buenaventura (Valle del Cauca) and Monteria (Cordoba) between January and December 2013. A sample was collected at the time of consultation for MFA. Parasitaemia was estimated by qPCR.

#### Blood samples

Whole blood (400 µL) was collected by venipuncture in tubes containing EDTA anticoagulant, and used as follows: 50 ml for TBS preparation, and a 200 µL aliquot which was added to 400 µl of Tempus RNA stabilization buffer (Applied Biosystems, UK) and preserved for molecular analysis at −20 °C. The remaining sample was immediately transported to the laboratory and placed at −20 °C.

### Assessment of *P. vivax* infectivity to mosquitoes

#### Mosquito membrane feeding assay (MFA)

Infected blood samples from acute and asymptomatic infected volunteers were sent at 37 °C from endemic areas to the nearest mosquito colony in Buenaventura or Monteria (<4 h in transit). Samples from experimentally infected volunteers was collected and tested in MFA at central laboratories in Cali within 4 h of collection. A laboratory-reared *Anopheles albimanus* mosquito colony was used for MFA as described before [17]. Briefly, blood samples (1 mL) were centrifuged at 500*g* for 3 min at 37 °C, packed red blood cells (RBCs) were washed with warm serum-free RPMI 1640 medium (Sigma, St Louis, MO, USA) and reconstituted to 50 % haematocrit using equal volumes of pooled AB+ non-immune human serum. For each assay, 200 female mosquitoes (3–4 days after emergence) were sugar and water starved overnight and fed with the reconstituted *P. vivax* field isolate.

#### Direct feeding assay (DFA)

For early infections, batches of 30 adult (3–4 days old) *An. albimanus* mosquitoes were placed in feeding boxes and were subject to overnight starvation prior to feeding. Fed mosquitoes were transported in cages to secure infection rooms and kept under strict security and laboratory conditions (constant 80 % humidity and 26 °C temperature). Infected volunteers were subjected to mosquitoes directly feeding on one arm and after 10 min of exposure, mosquitoes were transported and kept under safe and tightly controlled laboratory conditions [18].

### Assessment of *P. vivax* infectivity to mosquitoes

#### Microscopy

On day 7 post feeding, mosquitoes were dissected and examined microscopically at 40× for the presence of oocysts in the midgut after being stained with 2 % mercurochrome. Mosquito infections prevalence was expressed as infection percentage. Mosquito intensity was determined as the arithmetic mean of oocysts count per infected mosquito.

#### qPCR

High throughput methods for detecting infection in whole mosquitoes have shown that 18s PCR provides a reliable approximation of mosquito infection rates on day 7 post infection [19]. Molecular detection of *P. vivax* infections in mosquito midgut was performed by qPCR targeting the 18S ribosomal RNA gene. Primers and amplification conditions were described previously [15]. In summary, DNA was extracted from mosquito midguts after overnight proteinase K digestion al 56 °C in 180 μl of ATL buffer from QIAmp DNA Blood Mini Kit (QIAGEN, Valencia, CA, USA). Amplification qPCR was carried out in a total volume of 10 μL, containing 5 μL of TaqMan Universal Master Mix (Applied Biosystems, UK), 0.2 μL of each primer (10 nM stock), 0.2 μL Falcprobe (10 nM stock), and 0.2 μL Vivprobe (10 nM stock) and 2 μL of DNA. All amplification reactions were performed in 7500 Real-Time PCR Systems, (Applied Biosystems, USA).

### Quantitative reverse-transcriptase PCR assay

#### Primer design

In order to determine gametocyte stage, a quantitative reverse transcriptase PCR (RT-qPCR) was used to determine two sexual stage-specific molecular markers; *Pvs16* and *Pvs25*, which are *P. falciparum* orthologues known to define immature gametocytes and stage V female gametocytes, respectively [16]. The assay included primers targeting the markers described in Additional file 1: Table S1, which were designed by hand using the Primer Express software (Applied Biosystems) following recommended guidelines for qRT-PCR primer design. Primers were checked for homology against *Plasmodium* or human homologous sequences using PlasmoDB and NCBI Blast in order to eliminate the chances of non-specific amplification.

#### RNA extraction, DNase digest and reverse transcription

RNA was extracted from the 600 µl (200 µl whole blood +400 Tempus RNA stabilization buffer) aliquots conserved in Tempus RNA stabilization Buffer (Applied Biosystems, UK) using the PureLink RNA Mini Kit (Ambion, USA) following the manufacture conditions. Briefly, 200 µL of 1X PBS were added to the samples, mixed thoroughly using vortex, centrifuged at 12,000*g* for 30 min at 4 °C and the supernatant removed. After drying for 2 min, the pellet was suspended in 350 μL of lysis buffer and 350 μL of 70 % ethanol, and the whole volume transferred to a spin cartridge. Columns were centrifuged and washed with buffers one and two, RNA was eluted from the cartridge columns using Ultra-Pure water. RNA sample was added to 2 μL of DNase I, 2 μL of 10X DNase I buffer, and inactivated with 4 μL of DNase inactivation reagent. A second round of DNase digestion was performed to ensure genomic DNA removal. cDNA was obtained using a Super Script III Kit (Invitrogen, USA), according to the manufacturer’s instructions.

### Detection and quantification

Gene expression was performed in triplicate by RT-qPCR (7500 Real-Time PCR Systems; Applied Biosystems, USA) using SYBR Green and oligonucleotide primers as described (Additional file 1: Table S1). Two fragments of 351 and 423 base pairs of the pvs25 and Pvs16 mRNAs, respectively, were cloned into the pGEM-T Easy plasmid vector (Promega) which had been linearized by digestion with NotI (New England BioLabs). Triplicate spectrophotometry readings were used to determine DNA quantity and purity. Tenfold serial dilutions (6.2E^6–62 copies/µl) were PCR amplified in each test run and used to generate a standard curve. Data were analysed by the standard curve method of comparative quantification using standard curves of plasmid containing the target sequences [20]. Each reaction was performed in a total volume of 10 and 2 µL of cDNA. To ensure DNA elimination, each sample was tested by qPCR targeting the housekeeping *18S rRNA* gene of *P. vivax* in an independent reaction without reverse transcriptase. Sensitivity of this assay was determined using tenfold serial dilutions of cDNA. The melt curves were checked to control primer dimers. cDNA from malaria-negative individuals was used as negative amplification controls.

### Statistical analysis

The data were analysed using SPSS software version 19, IBM and Graph Pad Prism 6, GraphPad Prism^®^, the studied groups were compared by Kruskal–Wallis and Mann–Whitney tests. Differential expression analysis was performed using a one-way analysis of variance (ANOVA). For oocyst prevalence Fisher’s exact test was used. A p value <0.05 was considered statistically significant.

## Results

### Gametocyte maturation in early infection

**Fig. 1:**
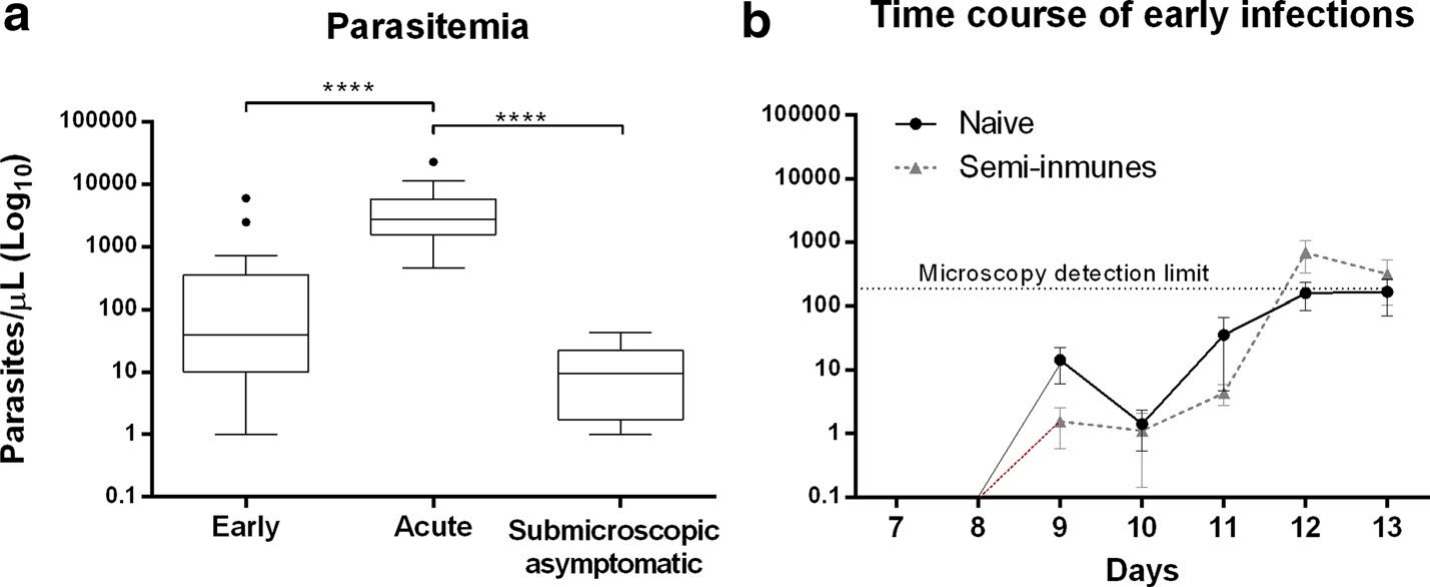
Parasitaemia distribution in the study groups. **a** Range comparison in the three groups; **b** Time course of early infections. The *error bars* represent the standard error of the mean (SEM), significant differences are indicated by an *asterisk* (p < 0.05 ANOVA followed by Tukey multiple comparison test)

The mean parasitaemia levels as determined by qPCR for early, acute and asymptomatic infections were 425.6 (1–6015), 5096 (459–22,800) and 13.93 (1–43), respectively, (Fig. 1a). Samples from volunteers experimentally infected with *P. vivax* sporozoites (early infections) were confirmed by qPCR to harbour *P. vivax* blood infection starting on day 7 after exposure to infected mosquitoes. PCR results confirmed blood infection in the two early infection sub-groups: semi-immune volunteers n = 9 (1–84 parasites/µL) and naïve volunteers n = 7 (57–105 parasites/µL) (Fig. 1b), although TBS remained negative until day 13 post infection.

Expression of gametocyte-stage specific genes was used to determine the course of gametocyte development and maturation. Gametocyte markers’ efficiency curves are shown in Additional file 2. Early infections displayed distinct patterns of sexually differentiated parasites, and a time-dependent increase of *Pvs16* and *Pvs25* expression. The slope was calculated as a measure of the transcription rate for both markers and the two slopes were compared to determine the relative speed of production. Although *Pvs25* transcripts were detected as early as day 7 after infection, the rate of *Pvs16* expression was almost 50 times faster than *Pvs25*. The *Pvs25/Pvs16* expression ratio was low at all time points (at day 13 the *Pvs25* transcripts mean was 27.5 copies/µL whereas *Pvs16* mean was 350 copies/µL).

### Early infected *P. vivax* carriers fail to infect *An. albimanus* mosquitoes

The infectivity of gametocytes from early infected volunteers to *An. albimanus* was assessed by both mosquito DFA and MFA performed simultaneously followed by evaluation of oocyst counts using both microscopy and qPCR, 7 days after experimental mosquito infection. Feeding assays were performed every 2 days from day 7 until day 13 when parasite patency was confirmed by TBS and volunteers received curative anti-malarial treatment. None of the samples obtained from individuals in early stages of infection (n = 16) showed infectivity to mosquitoes when assessed by either microscopy or PCR for the presence of oocysts.

Naturally infected symptomatic patients (n = 16) showed the presence of gametocytes (400–2820/µL) by TBS, and were additionally positive for the presence of gametocyte-specific transcripts corresponding to the following genes: *Pvs16* (mean 123 copies/µL) and *Pvs25* (mean 3473 copies/µL). Eight out of 16 symptomatic patients successfully infected mosquitoes with an oocyst average prevalence of 57 % as determined by MFA.

### Asymptomatic parasite carriers successfully infect *An. albimanus* mosquitoes

The prevalence of asymptomatic sub-microscopic *P. vivax* was 12.4 and 4.5 % in La Delfina and Zacarias, respectively. Individuals who did not present microscopically detectable parasites and showed no symptoms on the day of diagnosis were followed up for 7 days to confirm the asymptomatic condition. A sub-set of 14 asymptomatic volunteers was enrolled to assess the parasite transmission potential by MFA. Remarkably, 56 % of the asymptomatic carriers were infective to *An. albimanus* mosquitoes. Mosquitoes fed with blood from asymptomatic volunteers developed a low infection prevalence of 2.5 and 5 % from La Delfina and Zacarias, respectively, with oocyst loads ranging between one and ten per midgut (Table 1). This group of blood samples presented gametocyte markers as follows: *Pvs16* transcripts (mean 2395 copies/µL) and *Pvs25* transcripts (mean 46.30 copies/µL).

**Fig. 2:**
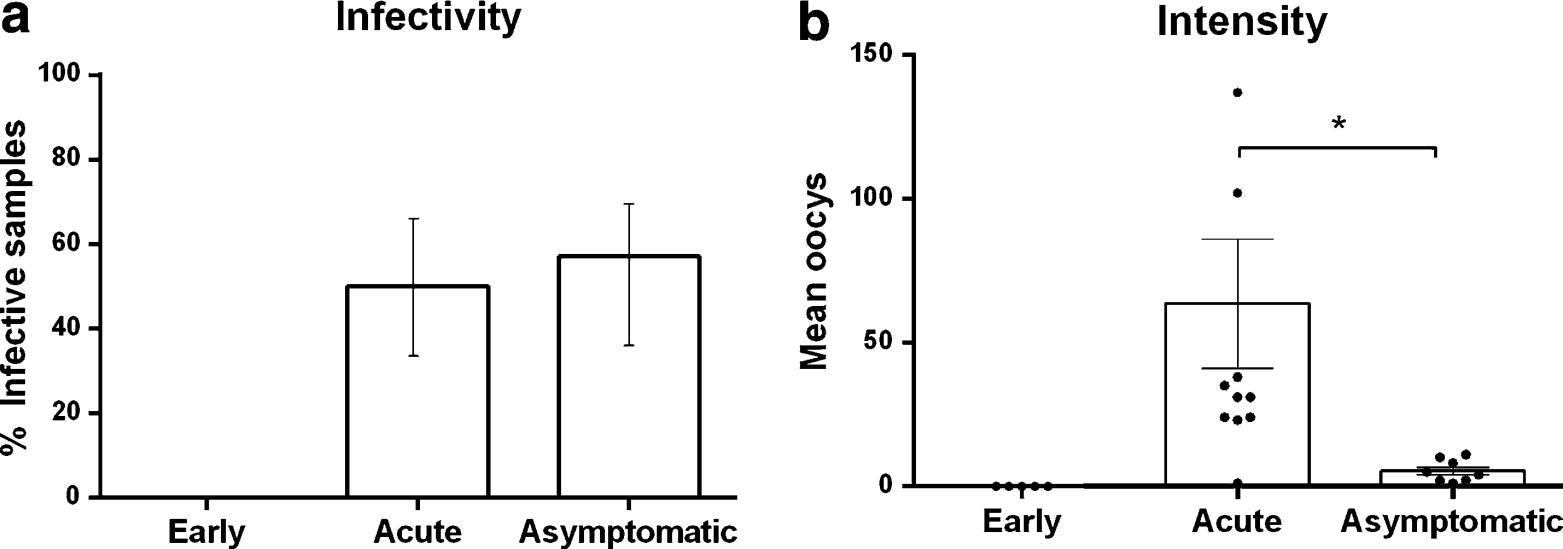
Infectivity to *Anopheles albimanus* mosquitoes. **a** Sample infectivity; **b** Oocyst intensity at the midgut

Despite the low parasitaemia levels (Fig. 2a), 57 % (8/14) samples from asymptomatic volunteers were infective to mosquitoes. The percentage of infective samples was similar in asymptomatic and acute volunteers although the latter displayed parasitaemia levels at least two orders of magnitude higher. Likewise, a significant difference was observed in the intensity of transmission between the asymptomatic and acute groups, with a mean of 5.4 and 63.5 oocysts, respectively, (Fig. 2a, b). Infective samples in asymptomatic patients had a high proportion of *Pvs25/Pvs16* in comparison with non-infective samples; however no statistically significant differences were observed (Additional file 3).

### *Plasmodium vivax* sexual development markers in early, acute and asymptomatic infections

**Fig. 3:**
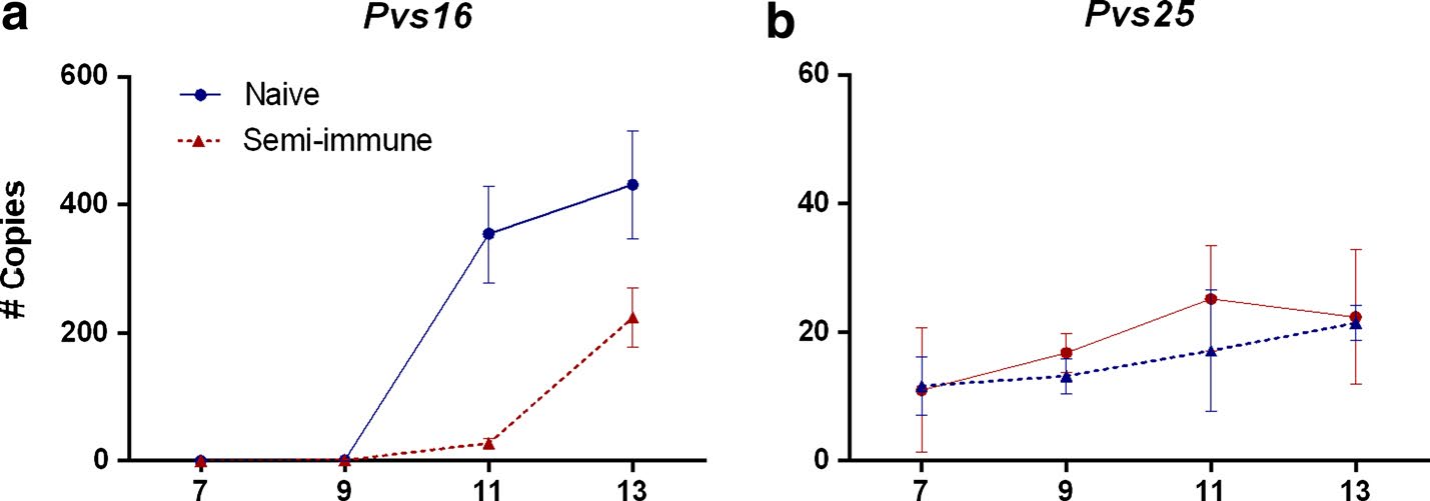
Relative expression of *Pvs16* (**a**), *Pvs25* (**b**), in early, acute and asymptomatic infections. *Each point* represents the expression against total parasites in Log10 scale. The *error bars* represent the SEM and significant differences are indicated by an *asterisk* (p < 0.05 ANOVA followed by Tukey multiple comparison test)

Relative expression of gametocyte markers (*Pvs16*, *Pvs25*) was determined using the 18s gene expression as reference. Expression of immature gametocyte marker *Pvs16* was significantly higher in asymptomatic volunteers (172.3) compared to relative expression from acute (0.019; p = 0.0049) and early infection samples (0.91; p = 0.0075). This trend was similar for the mature stage V gametocyte marker *Pvs25*, which showed a maximum relative expression in asymptomatic volunteers. Acute and early groups showed a low proportion of sexual stage parasites (mean ratio 0.58 for immature gametocytes and 0.25 for mature gametocytes) (Fig. 3a, b).

## Discussion

This study shows that parasites obtained from both *P. vivax* sub-microscopic asymptomatic individuals and naturally infected in the field were able to infect mosquitoes. Despite identification of *P. vivax* gametocytes in circulation as early as day 7 after experimentally induced infection, parasites were not infectious to mosquitoes when tested at days 7, 9, 11, and 13. Even at day 13, when parasites displayed the highest expression of mature gametocytes markers during the experimentally induced infection follow-up, they were still not infective.

Despite the fact that gametocytes commitment and differentiation start early on, during the initial phases parasites rapidly multiply, enlarging the parasite pool (asexual forms) and maintaining a low proportion of mature gametocytes, similar to the model of gametocyte sequestration in the bone marrow in *P. falciparum* [21]. On the other hand, in asymptomatic individuals, the mass of circulating parasites is mostly gametocytes. The absolute expression of *Pvs25* in parasites from asymptomatic volunteers was almost twice as much as in early infections, although parasitaemia was 20-fold lower.

Successful infections of *Anopheles* mosquitoes with blood from sub-microscopic asymptomatic carriers underscores the importance of targeting all parasite carriers as they should be considered potential malaria transmitters. In *P. falciparum* it has been experimentally shown that stage V gametocytes observed in both sub-microscopic and microscopic gametocytaemia contributed similarly to malaria transmission [22]. In regions with high malaria transmission, a significant proportion of the asymptomatic population harbours microscopically detectable parasitaemia. In Burkina Faso, an intense seasonal malaria transmission area, a recent study showed that *P. falciparum*-infected children were more important as the reservoir than adults and sub-microscopic infections [23]. In regions with low transmission intensity, a significantly higher number of cases are sub-microscopic [24]. A previous study in asymptomatic volunteers from Thailand using *P. vivax* and *An. dirus* mosquitoes resulted in 13 % of infective samples with low oocyst counts [25]. In this study, acute symptomatic infections presented high oocyst counts in mosquitoes, possibly due to the greater absolute numbers of mature gametocytes and adequate sex ratio; this appears to be in contrast with volunteers with asymptomatic infections, where the oocyst load was significantly lower. Although this is likely due to the low levels of mature gametocytes available, in terms of transmission maintenance the continuous presence of infected individuals and mosquitoes in endemic regions appears to be enough to maintain malaria spreading at community level. A study from Burkina Faso and Kenya on *P. falciparum* showed that low levels of gametocytes (<200/µL) are infective to *An. gambiae;* however, at higher densities a plateau region is reached [26]. In this study, even lower levels of mature *P. vivax* gametocytes are infective to mosquitoes in more than half of the cases.

The low proportion of mature gametocytes in acute and early infections in comparison with asymptomatic carriers appears to indicate that in early infections, the asexual parasite development and replication prevails in comparison to the sexual parasite development. However, the environmental changes that define the parasite cell fate remain largely unknown. The comparison between early, acute and asymptomatic infections could be useful for understanding the switch from asexual to sexual replication with potential applications towards development of novel drugs targeting malaria transmission.

Whereas it was possible precisely ascertain that gametocytes in experimentally induced infections corresponded to 13-day infections, this was not the case with naturally infected acute individuals. Early studies on neurosyphilitic patients using different *P. vivax* strains reported early successful feeds at days 7 and 10 of patency [4, 27]. Together, these results suggest that the infective patent period is modulated by the *P. vivax* strain, by multiple factors related to the human host [28], and possibly also the mosquito vector. Furthermore, parasite genetic variation, human immune response, and maturation and sexual distribution of gametocytes may be among these factors [6, 29].

**Fig. 4:**
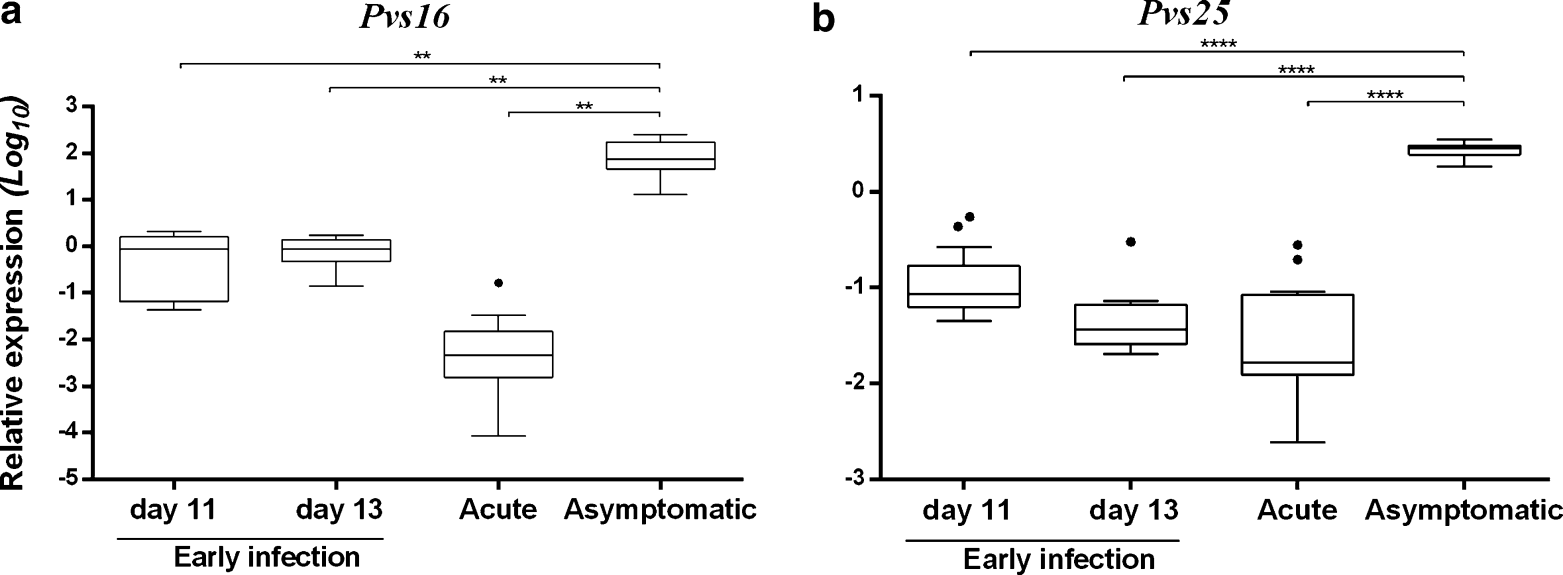
*Plasmodium vivax* sexual development in early infections. Number of copies of *Pvs16* (**a**), *Pvs25* (**b**) transcripts in experimentally infected volunteers during follow-up of the infectious challenge. The number of transcripts was determined from samples corresponding to days 7, 9, 11, and 13. Data shown are mean ± SD. *Asterisk P* value <0.05

An interesting observation here is that human immune response appears to influence gametocyte appearance. As shown in Fig. 4, infected naïve samples showed *Pvs25* expression earlier than those from semi-immune individuals who had experienced multiple previous malaria episodes and were known to have specific anti-*Plasmodium vivax* antibodies before experimental infection [14]. A better understanding of the role asymptomatic infections play in transmission is essential for implementation of improved malaria elimination programmes [24, 30–32]. Currently, the fact that asymptomatic carriers with low parasitaemias are not detected with the operative diagnosis methods (thick smears and RDTs) represents a great hurdle for malaria elimination.

## Conclusions

This study provides preliminary evidence on the *P. vivax* gametocyte maturation process and presents evidence supporting the role of asymptomatic sub-microscopic infections in malaria transmission. These results should be considered when new policies are envisioned to redirect malaria control strategies towards targeting asymptomatic infections as a tool for malaria elimination. Further longitudinal studies on asymptomatic volunteers, aimed at assessing roles of the host’s immune response and signalling pathways associated with the asymptomatic condition, are ongoing.

## Additional files

10.1186/s12936-016-1104-1 Primers used for amplification of *Pvs16*, *Pvs25*, orthologue genes using quantitative real-time RT-PCR.
10.1186/s12936-016-1104-1 Gametocyte markers efficiency curves: Efficiency of qRT-PCR reactions using 10-fold dilutions of cDNA pools . Efficiencies were in the range of (84 -97%) for all primers. R2- values were all >0.98.
10.1186/s12936-016-1104-1 Pvs25/Pvs16 correlation with infectivity. Samples from acutely infected patients were  compared by infectivity outcome. A trend was observed but no significant differences were found.

## References

[1] Gething PW, Elyazar IR, Moyes CL, Smith DL, Battle KE, Guerra CA (2012). A long neglected world malaria map: *Plasmodium vivax* endemicity in 2010. PLoS Negl Trop Dis.

[2] Krotoski W (1988). The hypnozoite and malarial relapse. Prog Clin Parasitol.

[3] Ankarklev J, Brancucci NM, Goldowitz I, Mantel P-Y, Marti M (2014). Sex: how malaria parasites get turned on. Curr Biol.

[4] Jeffery GM (1952). The infection of mosquitoes by *Plasmodium vivax* (Chesson strain) during the early primary parasitemias. Am J Trop Med Hyg.

[5] Contacos PG, Collins WE, Jeffery GM, Krotoski WA, Howard WA (1972). Studies on the characterization of *plasmodium vivax* strains from Central America. Am J Trop Med Hyg.

[6] Bousema T, Drakeley C (2011). Epidemiology and infectivity of *Plasmodium falciparum* and *Plasmodium vivax* gametocytes in relation to malaria control and elimination. Clin Microbiol Rev.

[7] Okell LC, Ghani AC, Lyons E, Drakeley CJ (2009). Submicroscopic infection in *Plasmodium falciparum*-endemic populations: a systematic review and meta-analysis. J Infect Dis.

[8] Noulin F, Borlon C, Van Den Abbeele J, D’Alessandro U, Erhart A (2013). 1912–2012: a century of research on *Plasmodium vivax* in vitro culture. Trends Parasitol.

[9] Bharti AR, Chuquiyauri R, Brouwer KC, Stancil J, Lin J, Llanos-Cuentas A (2006). Experimental infection of the neotropical malaria vector Anopheles darlingi by human patient-derived Plasmodium vivax in the Peruvian Amazon. Am J Trop Med Hyg.

[10] Abeles SR, Chuquiyauri R, Tong C, Vinetz JM (2013). Human host-derived cytokines associated with *Plasmodium vivax* transmission from acute malaria patients to Anopheles darlingi mosquitoes in the Peruvian Amazon. Am J Trop Med Hyg.

[11] Gonzalez-Ceron L, Rodriguez MH, Nettel JC, Villarreal C, Kain KC, Hernandez JE (1999). Differential susceptibilities of Anopheles albimanus and Anopheles pseudopunctipennis to infections with coindigenous *Plasmodium vivax* variants VK210 and VK247 in southern Mexico. Infect Immun.

[12] Laishram DD, Sutton PL, Nanda N, Sharma VL, Sobti RC, Carlton JM, Joshi H (2012). The complexities of malaria disease manifestations with a focus on asymptomatic malaria. Malar J.

[13] Vaccines mCGo (2011). A research agenda for malaria eradication: vaccines. PLoS Med.

[14] Arevalo-Herrera M, Forero-Pena DA, Rubiano K, Gomez-Hincapie J, Martinez NL, Lopez-Perez M, Castellanos A, Cespedes N, Palacios R, Onate JM, Herrera S (2014). Plasmodium vivax sporozoite challenge in malaria-naive and semi-immune Colombian volunteers. PLoS ONE.

[15] Rougemont M, Van Saanen M, Sahli R, Hinrikson HP, Bille J, Jaton K (2004). Detection of four Plasmodium species in blood from humans by 18S rRNA gene subunit-based and species-specific real-time PCR assays. J Clin Microbiol.

[16] Tao D, Ubaida-Mohien C, Mathias DK, King JG, Pastrana-Mena R, Tripathi A (2014). Sex-partitioning of the Plasmodium falciparum stage V gametocyte proteome provides insight into falciparum-specific cell biology. Mol Cell Proteomics.

[17] Hurtado S, Salas ML, Romero JF, Zapata JC, Ortiz H, Arevalo-Herrera M, Herrera S (1997). Regular production of infective sporozoites of *Plasmodium falciparum* and P. vivax in laboratory-bred Anopheles albimanus. Ann Trop Med Parasitol.

[18] Kone A, Mvd Vegte-Bolmer, Siebelink-Stoter R, van Gemert GJ, Dara A, Niangaly H (2010). Sulfadoxine–pyrimethamine impairs *Plasmodium falciparum* gametocyte infectivity and Anopheles mosquito survival. Int J Parasitol.

[19] Stone WJ, Eldering M, van Gemert G-J, Lanke KH, Grignard L, van de Vegte-Bolmer MG (2013). The relevance and applicability of oocyst prevalence as a read-out for mosquito feeding assays. Sci Rep.

[20] Pfaffl MW. AZ of Quantitative PCR, Chapter 3–Quantification strategies in real-time PCR. International University Line (IUL). La Jolla. Editors, SA Bustin. 2004.

[21] Joice R, Nilsson SK, Montgomery J, Dankwa S, Egan E, Morahan B (2014). *Plasmodium falciparum* transmission stages accumulate in the human bone marrow. Sci Transl Med.

[22] Schneider P, Bousema JT, Gouagna LC, Otieno S, van de Vegte-Bolmer M, Omar SA (2007). Submicroscopic Plasmodium falciparum gametocyte densities frequently result in mosquito infection. Am J Trop Med Hyg.

[23] Ouédraogo AL, Gonçalves BP, Gnémé A, Wenger EA, Guelbeogo MW, Ouédraogo A (2016). Dynamics of the human infectious reservoir for malaria determined by mosquito feeding assays and ultrasensitive malaria diagnosis in Burkina Faso. J Infect Dis.

[24] Coleman RE, Sattabongkot J, Promstaporm S, Maneechai N, Tippayachai B, Kengluecha A (2006). Comparison of PCR and microscopy for the detection of asymptomatic malaria in a *Plasmodium falciparum*/*vivax* endemic area in Thailand. Malar J.

[25] Coleman RE, Kumpitak C, Ponlawat A, Maneechai N, Phunkitchar V, Rachapaew N (2004). Infectivity of asymptomatic Plasmodium-infected human populations to Anopheles dirus mosquitoes in Western Thailand. J Med Entomol.

[26] Churcher TS, Bousema T, Walker M, Drakeley C, Schneider P, Ouédraogo AL (2013). Predicting mosquito infection from Plasmodium falciparum gametocyte density and estimating the reservoir of infection. Elife.

[27] McKenzie FE, Jeffery GM, Collins WE (2002). *Plasmodium vivax* blood-stage dynamics. J Parasitol.

[28] Mendis KN, David PH, Carter R (1990). Human immune responses against sexual stages of malaria parasites: considerations for malaria vaccines. Int J Parasitol.

[29] Reece SE, Drew DR, Gardner A (2008). Sex ratio adjustment and kin discrimination in malaria parasites. Nature.

[30] Baird J (1998). Age dependent characteristics of protection v. susceptibility to *Plasmodium falciparum*. Ann Trop Med Parasitol.

[31] Snow RW, Omumbo JA, Lowe B, Molyneux CS, Obiero J-O, Palmer A, Weber MW, Pinder M, Nahlen B, Obonyo C (1997). Relation between severe malaria morbidity in children and level of *Plasmodium falciparum* transmission in Africa. Lancet.

[32] Doolan DL, Dobaño C, Baird JK (2009). Acquired immunity to malaria. Clin Microbiol Rev.

