# Supplementary material for: Plasmodium vivax gametocyte infectivity in sub-microscopic infections
Source: Malar J. 2016 Jan 28;15:48. doi: 10.1186/s12936-016-1104-1 (PMC4730736; doi:10.1186/s12936-016-1104-1)
Supplement: Supplementary file 1 — 10.1186/s12936-016-1104-1 Primers used for amplification of Pvs16, Pvs25, orthologue genes using quantitative real-time RT-PCR. [file 12936_2016_1104_MOESM1_ESM.docx]

**Table S1:** Primers used for amplification of *Pvs16*, *Pvs25*, orthologue genes using quantitative real-time RT-PCR

| **Gen** | **ID** | **Primer** | | **PCR Efficiency** | **Detection limit (copy number)** |
| --- | --- | --- | --- | --- | --- |
| Pvs16 | XM_001613309.1 | F-TTCTAAGCAAAACAGACG | 93.2 | | 30 |
|  |  | R- ACACAAGAGGAGGATTAGG |  |  |  |
| Pvs25 | XM_001608410.1 | F- GACTTCATTATCTGTGTTAC | 94.1 | | 40 |
|  |  | R- TTGAGTCAGCCAGATGAG |  |  |  |
| Pv 18s | X13926 | F- GTTAAGGGAGTGAAGACGA TCAGA | 90.2 | | 40 |
|  |  | R-AACCCAAAGACTTTGATTTC TCATAA |  |  |  |
